# Write for Your Journal

**Published:** 1888-11

**Authors:** 


WRITE FOR YOUR JOURNAL.—We desire more of our subscribers to write for the RECORD. Practitioners in the country are not exempted from this request. Every doctor of experience knows something that others do not know. A good formula, a new thought, or new discovery on some point. One of the objects of journalism is to gather up all such discoveries, and publish them for the general good. It is thus that we may learn one from another, and thus that every one may do something to help his home journal and to push on the car of medical progress.

